# Investigating how blood cadmium levels influence cardiovascular health scores across sexes and dose responses

**DOI:** 10.3389/fpubh.2024.1427905

**Published:** 2024-08-21

**Authors:** Feng Chen, Hao Lin, Yuansi Zhang, Yu Zhang, Shaohe Chen

**Affiliations:** ^1^Department of Child Healthcare, Wenzhou People's Hospital, Wenzhou, China; ^2^Children’s Heart Center, The Second Affiliated Hospital and Yuying Children’s Hospital, Institute of Cardiovascular Development and Translational Medicine, Wenzhou Medical University, Wenzhou, China; ^3^Department of Gastroenterology, Pingyang Affiliated Hospital of Wenzhou Medical University, Wenzhou, China; ^4^Department of Traditional Chinese Medicine, Wenzhou Yebo Proctology Hospital, Wenzhou, China

**Keywords:** cadmium, cardiovascular health, sex factors, risk factors, NHANES

## Abstract

**Background:**

The association between exposure to cadmium (Cd) and cardiovascular health (CVH) has received considerable scientific interest. However, findings thus far have been inconclusive, particularly regarding sex-specific effects and dose–response relationships. The aim of our study was to investigate the relationships of blood Cd levels with the overall and component CVH scores.

**Methods:**

We used data from the 2011–2018 NHANES to assess CVH using indicators such as BMI, blood pressure, lipid profiles, glucose levels, diet, physical activity, nicotine use, and sleep quality, each rated on a 0–100 scale. The overall CVH score was calculated as the average of these indicators. We employed both multiple linear and restricted cubic spline analyses to examine the relationship between blood Cd levels and CVH scores, including nonlinear patterns and subgroup-specific effects.

**Results:**

Our analysis revealed that higher blood Cd levels were associated with lower overall CVH, nicotine exposure, sleep, and diet scores, with nonlinear decreases observed in overall CVH and nicotine exposure scores at specific thresholds (−1.447 and −1.752 log μg/dL, respectively). Notably, sex differences were evident; females experienced more adverse effects of Cd on CVH and lipid scores, while in males, Cd exposure was positively correlated with BMI, a link not observed in females.

**Conclusion:**

Our study highlights the complex interplay between blood Cd levels and various aspects of CVH, revealing significant dose–response relationships and sex disparities. These findings enhance our understanding of the biobehavioral mechanisms linking Cd exposure to cardiovascular risk.

## 1 Introduction

Cardiovascular disease (CVD) is the leading cause of death worldwide and has a major impact on both global health and the economy (1). In 2019, an estimated 523 million people were affected by CVD, resulting in 18.6 million deaths (2). As the incidence of CVD increases, effective prevention and management strategies are vital. In 2010, the American Heart Association (AHA) implemented the “Life’s Simple 7” (LS7) scoring system to measure cardiovascular health (CVH) based on seven essential components: body mass index, smoking status, diet, cholesterol levels, blood pressure, blood glucose levels, and physical activity (3). The scores from these components are combined to classify individuals into three CVH categories: poor, intermediate, or ideal. Research has consistently shown that higher CVH scores are associated with a lower incidence of CVD complications and mortality (4–7). In 2022, the AHA updated the LS7 to include sleep health, refining the scoring system to better reflect contemporary health challenges (8).

Recent research has also highlighted the significant role of environmental factors, such as cadmium (Cd) exposure, in CVD development (9). Cd, a common heavy metal detectable in blood, enters the human body through water, food, and air (10, 11). Recent studies have further emphasized the association between blood Cd levels and cardiovascular health outcomes. For instance, a comprehensive review indicates that elevated blood Cd levels are significantly associated with increased incidence and mortality rates of CVD, coronary artery disease, and stroke (12). Another study reported that higher blood Cd levels were associated with increased all-cause and cardiovascular mortality in patients with hypertension (13). Despite extensive studies, the relationship between Cd exposure and CVD remains unclear, particularly regarding sex-specific effects (14, 15). To address this research gap, we utilized the extensive data from the National Health and Nutrition Examination Survey (NHANES) spanning 2011 to 2018, setting clear research objectives. The primary goal is to thoroughly investigate the relationships between blood Cd levels and both overall and specific cardiovascular health scores. The secondary goal is to examine the potential influence of sex differences within these relationships. Our findings can inform more targeted and effective CVD prevention and management strategies.

## 2 Materials and methods

### 2.1 Study population

**Figure 1:**
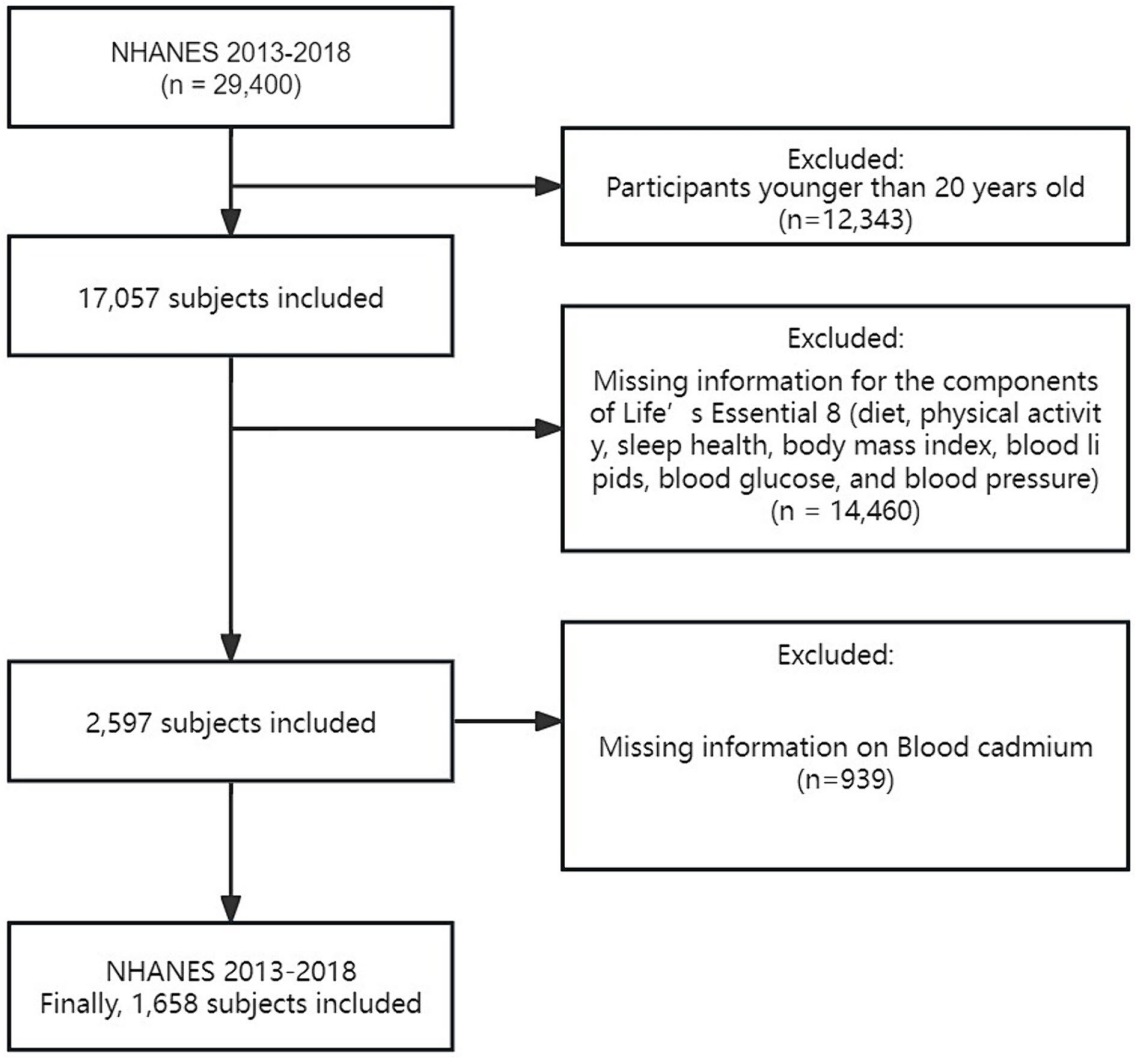
The study’s flow diagram.

We used data from the National Health and Nutrition Examination Survey (NHANES), which was conducted by the Centers for Disease Control and Prevention (CDC). The NHANES aims to assess the health and nutritional status of the U.S. population through interviews, biological samples, and physical exams. This comprehensive survey encompasses various aspects, such as interviews, biological sample collection, and physical examinations, to gather diverse health and nutrition-related data. Every individual involved in the study gave their informed consent, and the Institutional Review Board of the National Center for Health Statistics (NCHS) approved the research. Our analysis included data from three consecutive NHANES cycles (2013–2018), totaling 29,400 participants. We excluded individuals under 20 years old (*n* = 12,343), those missing key CVH component data (*n* = 14,460), and those lacking blood Cd measurements (*n* = 939). Our final sample consisted of 1,658 adults aged 20 and older with complete datasets for CVH score calculation and blood Cd levels (Figure 1). This sample includes both healthy individuals and those with various health conditions, reflecting a broad spectrum of cardiovascular health statuses.

### 2.2 Measurement of Cd

Blood Cd levels were measured using inductively coupled plasma mass spectrometry (ICP-MS) at the CDC’s National Center for Environmental Health. Samples were diluted with a solution containing tetramethylammonium hydroxide, EDTA, and other stabilizers, then atomized and ionized at high temperatures to quantify trace elements based on their mass-to-charge ratios. The detection limit was managed precisely, and for elements below this limit, values were imputed as the detection limit divided by the square root of two. Rigorous quality assurance protocols were followed to ensure the accuracy of the results.

### 2.3 CVH scores

CVH scores were based on eight components: blood pressure, BMI, blood glucose levels, blood lipid levels, physical activity, sleep duration, nicotine exposure, and diet. Each component was assessed on a 0–100 scale, as detailed in Supplementary Table S1 and the 2022 AHA Presidential Advisory (8). The composite CVH score aggregates these individual scores, categorizing CVH status into three levels: high (80–100), moderate (50–79), and low (0–49).

### 2.4 Covariates

Covariates were based on previous literature and assumptions related to CVH and included several factors (16–18): age, sex (female, male), race/ethnicity (Mexican American, non-Hispanic Black, non-Hispanic White, other race), educational attainment (less than high school, high school graduate, college or above), marital status (married/living with partner, divorced/widowed/separated, never married), poverty income ratio (PIR) (<1.3, ≥1.3), alcohol consumption status (<12 drinks/year, ≥12 drinks/year), waist circumference, and estimated glomerular filtration rate (eGFR). These variables were obtained through direct interviews or assessments of biological indicators. Alcohol consumption status was defined based on the question “Do you consume at least 12 alcoholic beverages per year?” The eGFR was calculated using the Chronic Kidney Disease Epidemiology Collaboration equation (19).

### 2.5 Statistical analyses

Descriptive statistics are presented as the mean ± standard deviation or median (interquartile range) for skewed distributions such as blood Cd, which were logarithmically transformed to achieve normality. Baseline characteristics across CVH categories and between sexes were compared using *t*-tests or Mann–Whitney U tests for continuous variables and chi–square tests for categorical variables. Both univariate and multivariate linear regression analyses explored the associations between blood Cd levels and CVH scores. Restricted cubic spline (RCS) curves were used to assess dose–response relationships, and threshold effect analysis was used to identify inflection points. Covariates adjusted in the models included sex, age, race, PIR, education, alcohol consumption, marital status, waist circumference, and eGFR. Sensitivity analyses, including subgroup analyses presented in forest plots and reanalyzes excluding participants with a history of CVD (n = 183), were conducted to confirm the robustness of our findings.

All analyses were performed using R Statistical Software (Version 4.3.1, The R Foundation)^1^ and the Free Statistical analysis platform (Version 1.9, Beijing, China). A two-tailed test was used, and a result was considered statistically significant when the *p*-value was <0.05.

## 3 Results

### 3.1 Population characteristics

Table 1 presents the basic characteristics of the study population classified by CVH. The study involved 1,658 individuals, with an average age of 49.5 ± 17.4 years. Among them, 61.9% were male, and 38.1% were female. Male sex, higher economic income and education levels, non-Hispanic white race, married/cohabitating status, a higher eGFR, and a smaller waist circumference were associated with a higher CVH score. Importantly, participants with high CVH scores had lower blood Cd concentrations (*p* < 0.05). Additionally, we compared baseline data between different sexes. Compared to male participants, female participants exhibited higher blood Cd concentrations (*p* < 0.05) (Supplementary Table S3).

**Table 1:** Baseline characteristics of participants classified by their overall CVH score.

Characteristic	Total (*n* = 1,658)	Low CVH (*n* = 163)	Moderate CVH (*n* = 1,232)	High CVH (*n* = 263)	*p-*value
Sex^a^					< 0.001
Male	1,026 (61.9)	96 (58.9)	793 (64.4)	137 (52.1)	
Female	632 (38.1)	67 (41.1)	439 (35.6)	126 (47.9)	
Age^a^, years	49.5 ± 17.4	48.5 ± 14.5	49.8 ± 17.6	48.7 ± 18.0	0.471
Race^a^					< 0.001
Mexican American	160 (9.7)	17 (10.4)	128 (10.4)	15 (5.7)	
Non-Hispanic White	764 (46.1)	58 (35.6)	569 (46.2)	137 (52.1)	
Non-Hispanic Black	351 (21.2)	55 (33.7)	271 (22)	25 (9.5)	
Other race	383 (23.1)	33 (20.2)	264 (21.4)	86 (32.7)	
Family poverty-income ratio^a^					< 0.001
<1.3	432 (26.1)	58 (35.6)	340 (27.6)	34 (12.9)	
≥1.3	1,226 (73.9)	105 (64.4)	892 (72.4)	229 (87.1)	
Educational level^a^					< 0.001
Below high school	265 (16.0)	35 (21.5)	204 (16.6)	26 (9.9)	
High-school graduate	392 (23.6)	55 (33.7)	301 (24.4)	36 (13.7)	
College or above	1,001 (60.4)	73 (44.8)	727 (59)	201 (76.4)	
MS, (%)^a^					0.012
Married/living with partner	967 (58.3)	88 (54)	700 (56.8)	179 (68.1)	
Widowed/divorced/separated	378 (22.8)	40 (24.5)	293 (23.8)	45 (17.1)	
Never married	313 (18.9)	35 (21.5)	239 (19.4)	39 (14.8)	
Drinking status, (%)^a^					0.33
No	776 (53.3)	65 (48.5)	576 (53.2)	135 (56.5)	
Yes	680 (46.7)	69 (51.5)	507 (46.8)	104 (43.5)	
Waist circumference, cm^a^	100.0 ± 16.1	113.5 ± 16.9	100.7 ± 15.3	88.5 ± 10.4	< 0.001
eGFR, mL/min/1.73 m^2^^a^	96.7 ± 22.9	97.6 ± 22.4	95.8 ± 23.1	100.6 ± 21.6	0.007
log Cd, log μg/dL^a^	−0.8 ± 0.9	−0.5 ± 0.9	−0.8 ± 0.9	−1.1 ± 0.8	< 0.001

CVH, cardiovascular health; Low CVH 0–49; Moderate CVH 50–79; High CVH 80–100.

a Continuous variables are presented as mean ± SD; categorical variables are presented as N(%).

### 3.2 Associations between overall and component CVH scores and blood Cd concentrations

The linear model relationships between the overall and component CVH scores and the ln-transformed blood Cd concentrations (continuous and categorized) are presented in Table 2. According to the adjusted multivariate model, the ln-transformed blood Cd concentration was negatively associated with the overall score, nicotine exposure score, sleep score, and diet score (overall score: β = −3.32, 95% CI: −3.97 to −2.67; nicotine exposure score: β = −19.41, 95% CI: −20.94 to-17.88; sleep score: β = −1.99, 95% CI: −3.5 to −0.47; diet score: β = −5.65, 95% CI: −8.02 to −3.27). When blood Cd concentrations were converted into four categorical variables using the first quartile as the reference and adjusted for multiple variables, consistent results were observed, confirming the impact of excessive Cd levels on the overall and component CVH scores.

**Table 2:** Association between the blood Cd concentrations and CVH scores.

		CVH		Body mass index		Blood pressure		Blood lipids		Blood glucose		Physical activity		Nicotine exposure		Sleep health		Diet	
		Model 1	Model 2	Model 1	Model 2	Model 1	Model 2	Model 1	Model 2	Model 1	Model 2	Model 1	Model 2	Model 1	Model 2	Model 1	Model 2	Model 1	Model 2
Cd																			
Continuous	1,658	−2.95 (−3.66 ~ −2.25)*	−3.32 (−3.97 ~ −2.67)*	3.43 (1.58 ~ 5.27)*	0.19 (−0.92 ~ 1.29)	0.48 (−1.43 ~ 2.39)	0.35 (−1.54 ~ 2.23)	0.13 (−1.57 ~ 1.83)	−1.28 (−3.12 ~ 0.56)	1.41 (−0.08 ~ 2.9)	0.53 (−0.9 ~ 1.97)	−0.21 (−1.22 ~ 0.8)	0.67 (−0.46 ~ 1.79)	−20.3 (−21.98 ~ −18.62)*	−19.41 (−20.94 ~ −17.88)*	−2.67 (−4.04 ~ −1.31)*	−1.99 (−3.5 ~ −0.47)*	−5.89 (−8.16 ~ −3.62)*	−5.65 (−8.02 ~ −3.27)*
Q1	398	Ref	Ref	Ref	Ref	Ref	Ref	Ref	Ref	Ref	Ref	Ref	Ref	Ref	Ref	Ref	Ref	Ref	Ref
Q2	413	0.19 (−1.54 ~ 1.92)	−1.25 (−2.78 ~ 0.28)	2.61 (−1.95 ~ 7.18)	−0.24 (−2.84 ~ 2.35)	0.99 (−3.73 ~ 5.72)	3.77 (−0.66 ~ 8.21)	−0.9 (−5.1 ~ 3.31)	−0.82 (−5.15 ~ 3.52)	−1.45 (−5.12 ~ 2.23)	0.47 (−2.92 ~ 3.85)	−1.14 (−3.64 ~ 1.36)	−0.95 (−3.6 ~ 1.7)	−7.2 (−11.27 ~ −3.13)	−14.07 (−17.67 ~ −10.48)*	2.01 (−1.36 ~ 5.39)	0.61 (−2.97 ~ 4.18)	6.55 (0.98 ~ 12.13)*	1.22 (−4.38 ~ 6.81)
Q3	430	−2.39 (−4.1 ~ −0.68)*	−4.33 (−5.89 ~ −2.77)*	6.67 (2.15 ~ 11.19)*	−1.16 (−3.8 ~ 1.48)	−1.7 (−6.38 ~ 2.97)	0.34 (−4.17 ~ 4.86)	1.64 (−2.52 ~ 5.81)	0.34 (−4.07 ~ 4.75)	1.08 (−2.55 ~ 4.72)	1.95 (−1.49 ~ 5.39)	−0.26 (−2.74 ~ 2.21)	0.04 (−2.66 ~ 2.73)	−21.19 (−25.22 ~ −17.16)*	−25.78 (−29.44 ~ −22.11)*	−2.31 (−5.65 ~ 1.03)	−3.43 (−7.07 ~ 0.21)	−3.05 (−8.57 ~ 2.47)	−6.94 (−12.64 ~ −1.25)*
Q4	417	−6.69 (−8.42 ~ −4.97)*	−7.67 (−9.26 ~ −6.08)*	7.84 (3.29 ~ 12.39)*	0.43 (−2.27 ~ 3.13)	2.21 (−2.51 ~ 6.92)	0.97 (−3.65 ~ 5.59)	−0.03 (−4.23 ~ 4.16)	−3.75 (−8.26 ~ 0.76)	3.49 (−0.18 ~ 7.15)	0.85 (−2.66 ~ 4.37)	−0.78 (−3.28 ~ 1.71)	1.11 (−1.64 ~ 3.87)	−48.68 (−52.74 ~ −44.62)*	−46.29 (−50.03 ~ −42.55)*	−5.02 (−8.39 ~ −1.65)*	−3.36 (−7.08 ~ 0.36)	−12.55 (−18.11 ~ −6.99)*	−11.32 (−17.14 ~ −5.5)*

CVH, cardiovascular health; Model 1 was crude model; Model 2 was adjusted for age, sex, race, family PIR, educational level, marital status, drinking status, waist circumference, and eGFR. *P < 0.05.

**Figure 2:**
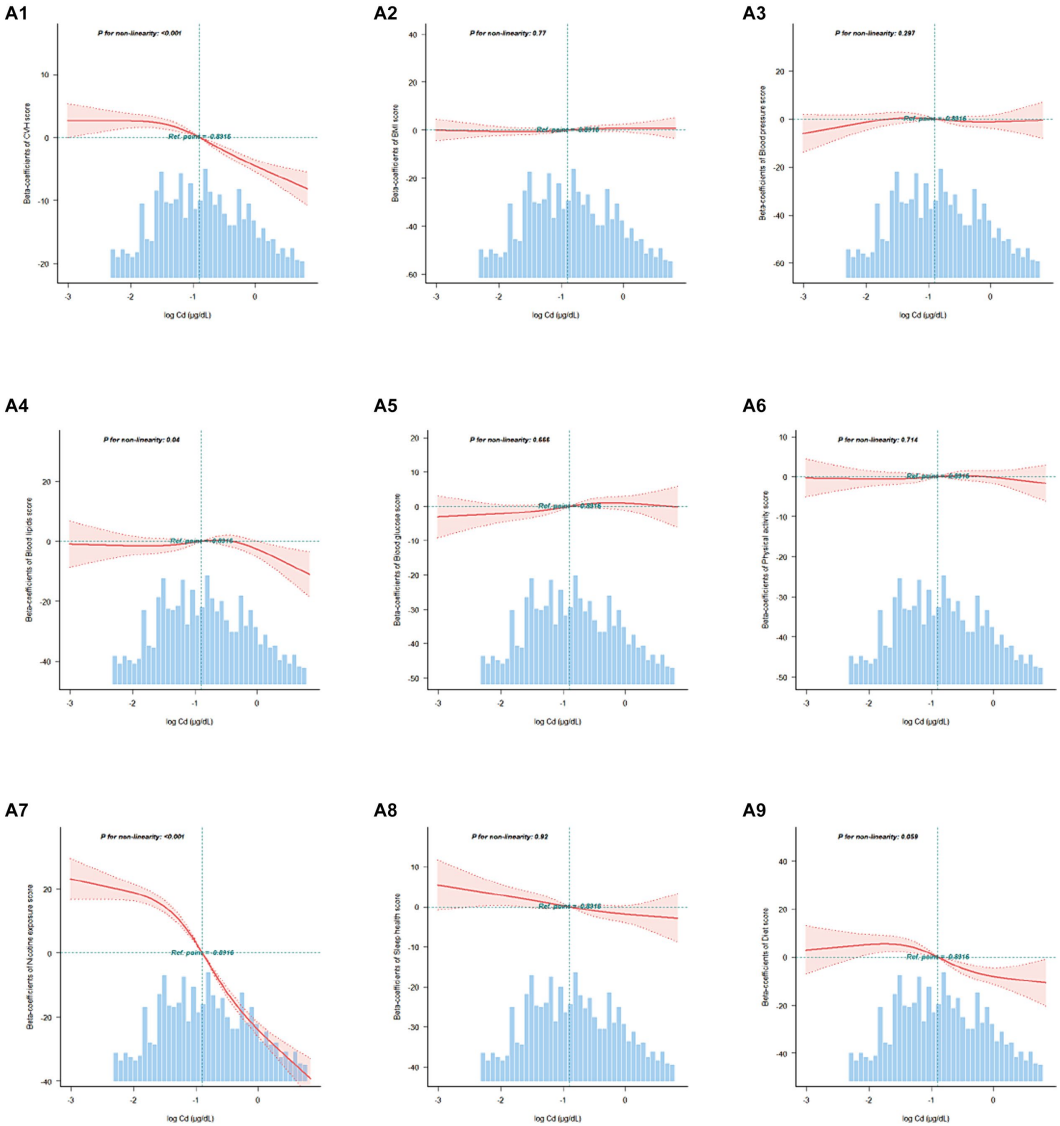
Association between the overall and component CVH scores and blood Cd levels beta-coefficients. Solid and dashed lines represent the predicted value and 95% confidence intervals. The models were adjusted for age, sex, race, family PIR, educational level, marital status, drinking status, waist circumference, and eGFR. A1, CVH scores; A2, Body mass index scores; A3, Blood pressure scores; A4, Blood lipids scores; A5, Blood glucose scores; A6, Physical activity scores; A7, Nicotine exposure scores; A8, Sleep health scores; A9, Diet scores.

In addition, RCS curves demonstrated the dose–response relationship between the overall and component CVH scores and blood Cd concentrations (Figure 2). We found a nonlinear correlation between the blood Cd concentration and the overall score and between the blood Cd concentration and the nicotine exposure score (p for nonlinearity <0.05). Threshold effect analysis indicated that when the blood Cd concentration reached approximately −1.447 log μg/dL, the CVH rapidly decreased, and when the blood Cd concentration reached approximately −1.752 log μg/dL, the nicotine exposure score also rapidly decreased (Supplementary Tables S4, S5).

### 3.3 Sensitivity analyses

**Figure 3:**
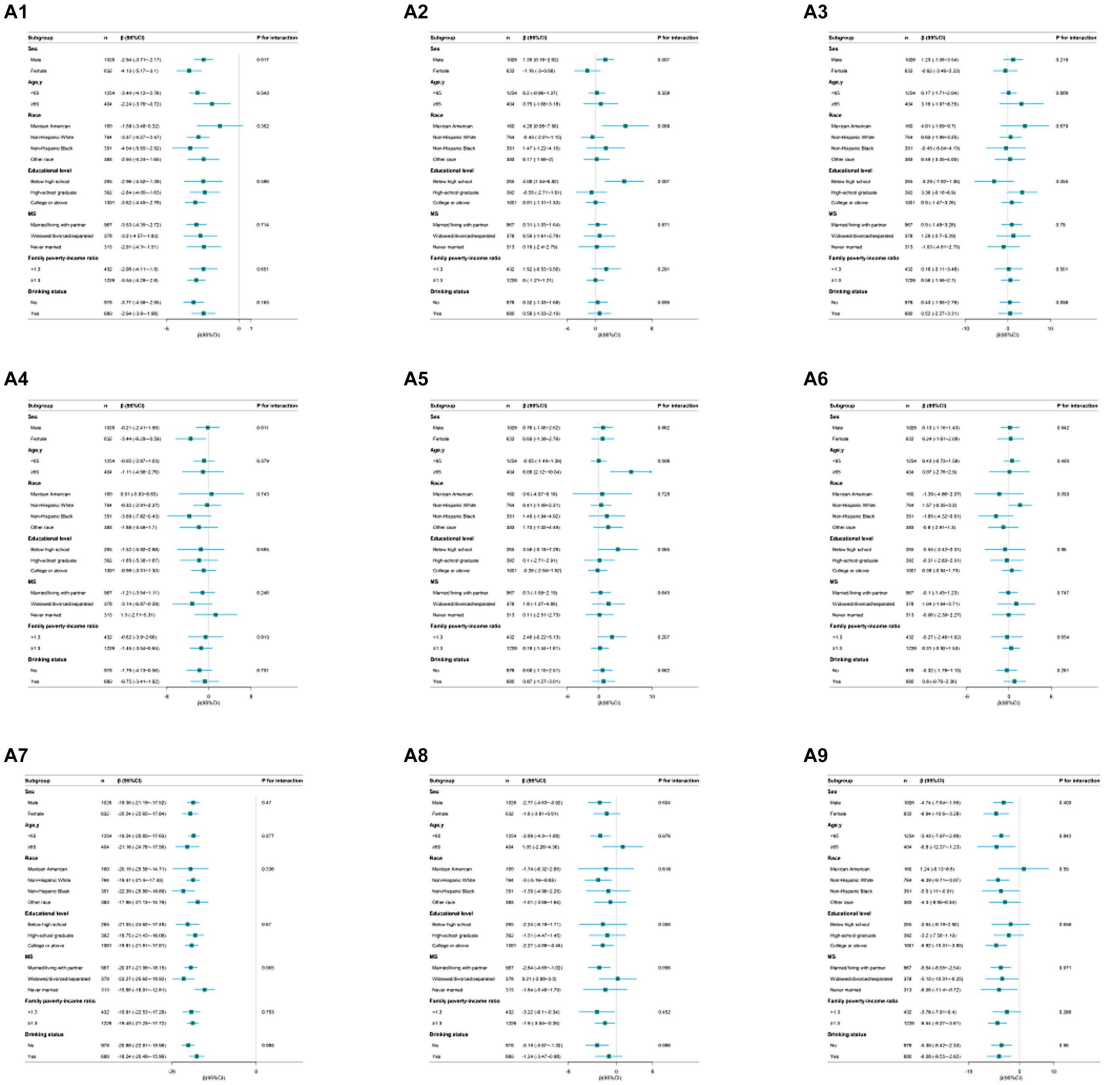
Subgroup analysis of the association between the overall and component CVH scores and blood Cd levels. Each stratification was adjusted for age, sex, race, family PIR, educational level, marital status, drinking status, waist circumference, and eGFR. A1, CVH scores; A2, Body mass index scores; A3, Blood pressure scores; A4, Blood lipids scores; A5, Blood glucose scores; A6, Physical activity scores; A7, Nicotine exposure scores; A8, Sleep health scores; A9, Diet scores. Except the stratification factor itself. Squares indicate β, with horizontal lines indicating 95% CIs.

Figure 3 shows consistent correlations between blood Cd concentrations, CVH scores, and various subscale scores across age, race, education level, income, marital status, and alcohol consumption. However, there was a significant interaction effect among sex groups. Specifically, among females, the ln-transformed blood Cd levels and the total CVH score and lipid score displayed stronger negative correlations. On the other hand, ln-transformed blood Cd levels were positively correlated with BMI in males but not in females (Supplementary Figures S1, S2).

Additionally, after excluding individuals with a history of CVD, the associations between the blood Pb level and the overall CVH score remained consistent, while other results showed a similar impact of blood Cd levels on CVH scores (Supplementary Table S6).

## 4 Discussion

In this study, we utilized NHANES data from 2011 to 2018 waves to investigate the relationships between the blood concentrations of Cd and overall and component CVH scores. We found a complex and rational relationship among them. Additionally, we observed sex differences in the effects of Cd exposure.

Over the past century, we have observed a notable increase in environmental pollution and consequent human exposure to Cd (20). The most common sources of Cd contamination are waste, industrial emissions, and soil, which lead to ingestion via food, tobacco smoke, and occupational hazards (11). Prospective studies have established a link between Cd concentrations and adverse CVD outcomes (21–23), supported by evidence that Cd-induced endothelial dysfunction could accelerate atherosclerosis (24). Our findings align with these scholarly insights (25–27), emphasizing the need for stringent monitoring of environmental Cd. Intriguingly, we identified a nonlinear relationship between blood Cd levels and overall CVH scores, with a marked decline in overall CVH scores at a blood Cd concentration of-1.447 log μg/dL, akin to the findings of Tellez-Plaza et al. (21). They found that urinary Cd levels above 0.57 μg/g were associated with increased CVD mortality. Another study found that the exposure-response relationship between blood Cd levels and acute coronary events appears to be relatively linear up to a blood Cd level of 1 μg/L, after which it levels off (28), suggesting a potential benchmark for safe blood Cd ranges. We corroborated this threshold and recommend that it be considered in future health guidelines. The biological effects of Cd indicate that at low exposure levels, the body might mitigate oxidative stress through natural antioxidant systems, thereby not exhibiting significant toxic effects. However, when Cd exposure exceeds the processing capacity of the body’s antioxidant systems, these systems may be overwhelmed, leading to a sharp increase in intracellular oxidative stress and consequently causing extensive damage to cell structure and function (29).

Additionally, our subgroup analysis showed significant sex differences in the association between blood Cd levels and CVH scores, with a stronger negative association observed in females. This finding is consistent with existing research, where multiple studies have shown correlations between Cd concentrations in the blood and urine of adult females and increased incidences of peripheral arterial disease, myocardial infarction, and increased intima-media thickness of the carotid arteries, whereas these correlations were not found in males (24, 30, 31). Studies suggest that females generally have higher levels of Cd in their bodies (32). Women might absorb more Cd through the gastrointestinal tract, where Cd enters the body, binds with metallothionein, and then accumulates in other significant organs and tissues, eliciting a more intense inflammatory response (33, 34). In addition to increased bodily Cd levels, females may exhibit increased expression of the metallothionein IIA gene (35). Animal studies have shown that Cd exposure increases the reactivity of male rats’ blood vessels to norepinephrine, leading to elevated blood pressure (36). Another study revealed that Cd had a reduced lethal effect on ovariectomized female rats, indicating that estrogen plays a role in the response to Cd exposure (37), which might explain the differences in Cd toxicity related to cardiovascular health between males and females. However, other scholars have reached differing conclusions, with environmental Cd exposure correlating with increased CVD mortality rates in males but not in females (22). Another study indicated that blood Cd levels are positively correlated with the 10-year risk score for atherosclerotic cardiovascular disease (ASCVD), with the risk significantly increasing in populations with higher blood Cd levels, particularly among men (38). Therefore, future research needs to further explore how sex differences affect the impact of Cd exposure on cardiovascular health. We also found that blood Cd levels were negatively correlated with nicotine exposure, sleep, and diet. This is likely because smoking is a primary pathway for Cd exposure (39). Moreover, recent research has shown that Cd can disrupt sleep patterns by causing sleep interruptions and decreasing the length of rapid eye movement (REM) sleep stages (40). Research by Unno et al. has shown that Cd in drinking water induces oxidative stress, resulting in an elevation of non-REM sleep levels and a reduction in rhythmic physical activity (41). Thus, the findings of previous studies confirming the impact of Cd on overall sleep quality are consistent with our findings. Additionally, Cd is commonly found in staple foods such as leafy vegetables and grains (11). Participants with higher blood Cd levels tended to have a diet richer in refined grains, which can reasonably explain our conclusions (Supplementary Table S7). Although no correlation was found between blood Cd levels and lipid scores in the overall population, our subgroup analysis revealed a significant negative correlation between these variables in female participants—a rare observation. This finding contrasts with studies that did not stratify by sex and found no correlation between low-level Cd exposure and lipid levels (42). A previous study involving Korean adults revealed that the blood Cd concentration was positively correlated with the risk of low-density lipoprotein cholesterol in a dose-dependent manner (43). The sex differences were notable: for males, high blood Cd was associated with an increased risk of low HDL-C and a high triglyceride-to-HDL-C ratio; for females, this association was weaker, potentially due to ethnic differences. Further research is required to confirm these findings. Another significant observation is that blood Cd levels in males were positively correlated with BMI, indicating a connection between higher Cd levels and lower BMI—this was not observed in females. A study on Chinese adults revealed that blood Cd concentrations were negatively associated with overweight status, with no sex differences observed (44). Another prospective cohort study in Mexico revealed that the effects of prenatal exposure to Cd continues into adolescence, affecting obesity, and this relationship was observed only in girls, not boys (45). The differences in outcomes might be related to Cd’s endocrine-disrupting properties affecting fat distribution. Laboratory studies have revealed interactions between Cd and estrogen and between Cd and androgen receptors, which activate estrogen receptor alpha (46). The lipid mobilization and fat breakdown in the body are regulated by hormone receptors (47), and this estrogenic effect might account for the inverse relationship with body fat seen in females. Conversely, exposure to Cd in males was linked to reduced levels of estradiol and testosterone in circulation, potentially elucidating the sex-specific outcomes (48). Therefore, when assessing the impact of blood Cd on body weight, these sex differences should be considered.

Our study has several advantages. First, the NHANES database contains a large amount of detailed information about a diverse population in the United States. The database is maintained with consistent data collection techniques and rigorous quality control measures to guarantee the precision and dependability of the data. Second, the latest CVH scores include behaviors and factors that impact CVH. These scores are currently the most advanced, and through our comprehensive evaluation, they have promoted new perspectives for understanding the effects of Cd on CVH. Several limitations must be taken into account. First, the cross-sectional nature of this database prevents us from establishing causal relationships; we can only infer correlations. Second, despite controlling for various confounding variables, there may still be unmeasurable factors that could confound the results. Third, due to potential variations in Cd exposure environments and differences in dietary and lifestyle habits among different countries and populations, our conclusions may not be generalizable to other countries or populations.

## 5 Conclusion

In conclusion, our research revealed complex and multifaceted associations of blood Cd levels with overall and component CVH scores. Notably, we identified nonlinear correlations of blood Cd levels with the overall CVH score and nicotine exposure score, with critical thresholds. Furthermore, sex differences were observed in the effects on blood Cd levels. Subsequent studies might also explore potential preventive and therapeutic interventions, refine risk assessment models, and extend these findings to broader populations to cement our understanding of these relationships.

## Data Availability

Publicly available datasets were analyzed in this study. This data can be found at: https://wwwn.cdc.gov/nchs/nhanes/Default.aspx.

## Footnotes

^1^ http://www.R-project.org

## Supplementary material

Supplementary Figure S1 Association between the male overall and component CVH scores and blood Cd levels beta-coefficients. Solid and dashed lines represent the predicted value and 95% confidence intervals. The models were adjusted for age, sex, race, family PIR, educational level, marital status, drinking status, waist circumference, and eGFR. A1, CVH scores; A2, Body mass index scores; A3, Blood pressure scores; A4, Blood lipids scores; A5, Blood glucose scores; A6, Physical activity scores; A7, Nicotine exposure scores; A8, Sleep health scores; A9, Diet scores.

Supplementary Figure S2 Association between the female overall and component CVH scores and blood Cd levels beta-coefficients. Solid and dashed lines represent the predicted value and 95% confidence intervals. The models were adjusted for age, sex, race, family PIR, educational level, marital status, drinking status, waist circumference, and eGFR. A1, CVH scores; A2, Body mass index scores; A3, Blood pressure scores; A4, Blood lipids scores; A5, Blood glucose scores; A6, Physical activity scores; A7, Nicotine exposure scores; A8, Sleep health scores; A9, Diet scores.
