# Supplementary figures and images for: Investigating how blood cadmium levels influence cardiovascular health scores across sexes and dose responses
Source: Front Public Health. 2024 Aug 21;12:1427905. doi: 10.3389/fpubh.2024.1427905 (PMC11371710; doi:10.3389/fpubh.2024.1427905)

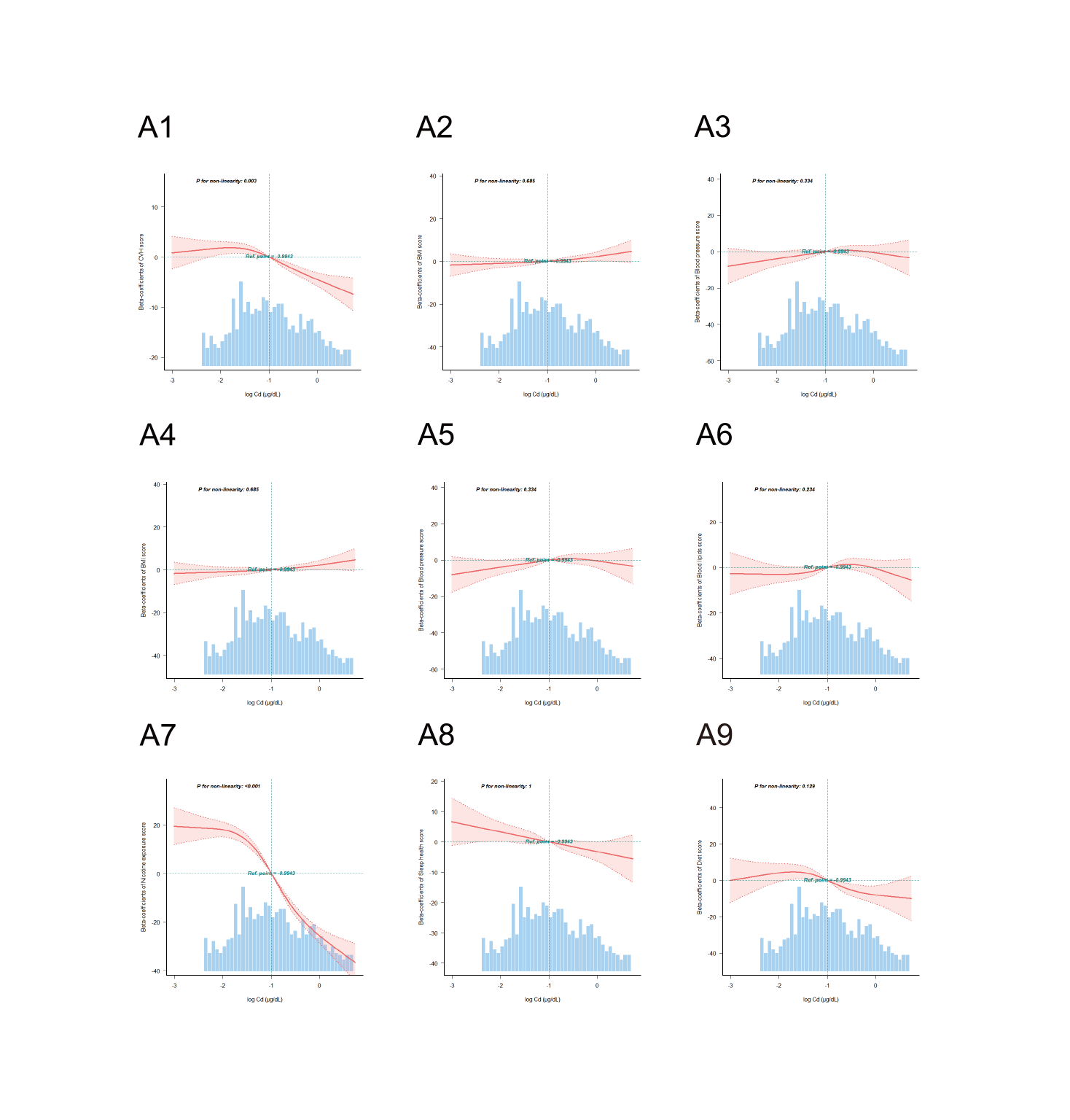

Supplement: Supplementary Figure S1 — Association between the male overall and component CVH scores and blood Cd levels beta-coefficients. Solid and dashed lines represent the predicted value and 95% confidence intervals. The models were adjusted for age, sex, race, family PIR, educational level, marital status, drinking status, waist circumference, and eGFR. A1, CVH scores; A2, Body mass index scores; A3, Blood pressure scores; A4, Blood lipids scores; A5, Blood glucose scores; A6, Physical activity scores; A7, Nicotine exposure scores; A8, Sleep health scores; A9, Diet scores. [file Figure_1.TIF]

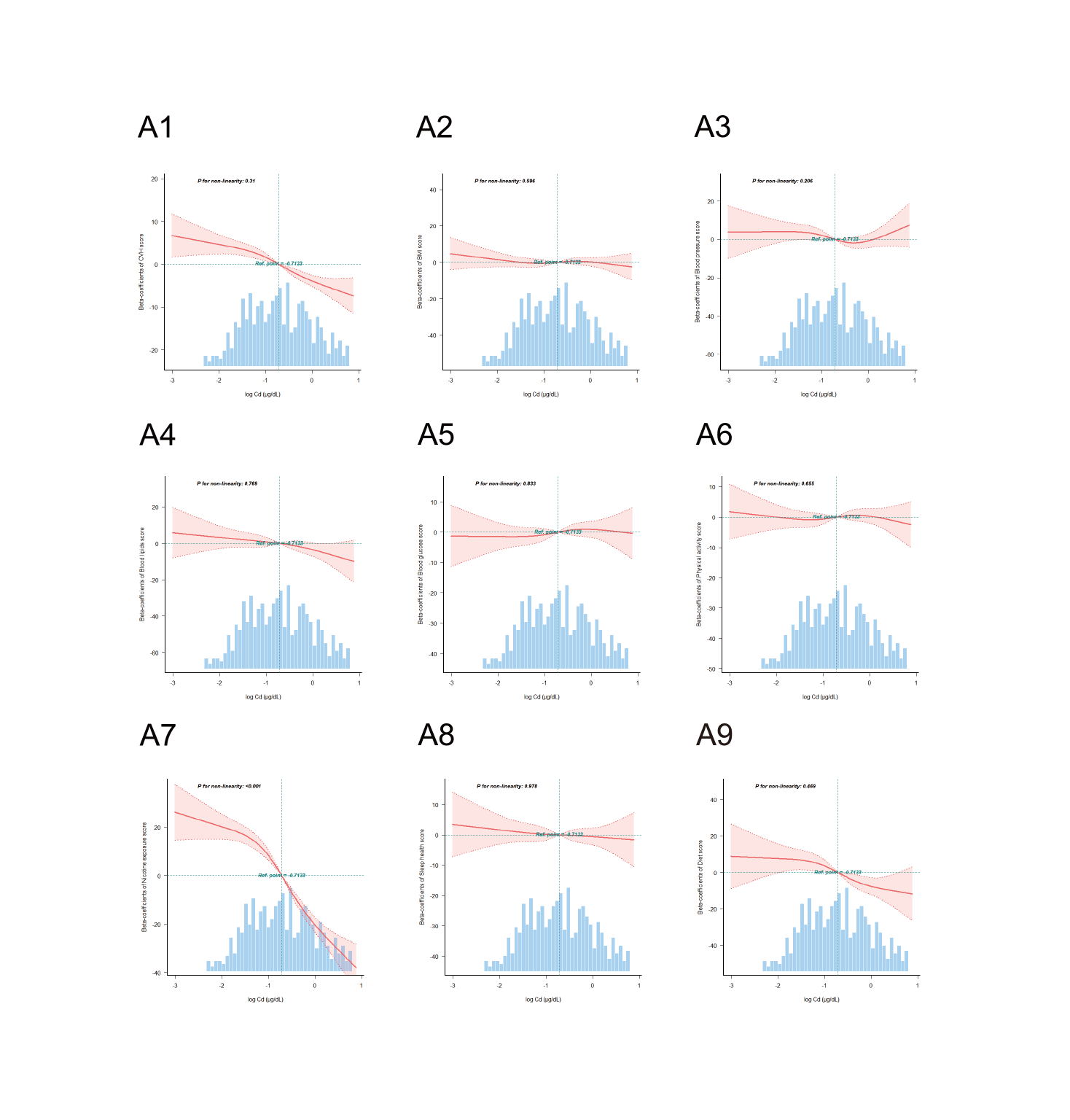

Supplement: Supplementary Figure S2 — Association between the female overall and component CVH scores and blood Cd levels beta-coefficients. Solid and dashed lines represent the predicted value and 95% confidence intervals. The models were adjusted for age, sex, race, family PIR, educational level, marital status, drinking status, waist circumference, and eGFR. A1, CVH scores; A2, Body mass index scores; A3, Blood pressure scores; A4, Blood lipids scores; A5, Blood glucose scores; A6, Physical activity scores; A7, Nicotine exposure scores; A8, Sleep health scores; A9, Diet scores. [file Figure_2.TIF]
