# Supplementary material for: Investigating how blood cadmium levels influence cardiovascular health scores across sexes and dose responses
Source: Front Public Health. 2024 Aug 21;12:1427905. doi: 10.3389/fpubh.2024.1427905 (PMC11371710; doi:10.3389/fpubh.2024.1427905)
Supplement: Supplementary file 3 [file Table_1.DOCX]

**Table S1** Life’s Essential 8 scoring rules

| **Domain** | **CVH metric** | **Method of measurement** | **Quantification of CVH metric** |
| --- | --- | --- | --- |
| Health behaviors | Diet | Dietary intake data were collected from NHANES participants through up to two 24-hour dietary recalls, in person during the first recall and by telephone during the second recall 3-10 days later. Applying HEI-2015 to Evaluate Dietary Quality(Table S2). | Points: HEI-2015 score:  100 ≥95th percentiles  80 75th-94th percentiles  50 50th-74th percentiles  25 25th-49th percentiles  0 1st-24th percentiles |
|  | Physical activity | Self-reported minutes of  moderate or vigorous PA  per week | Points: Minutes:  100 ≥150  90 120–149  80 90–119  60 60–89  40 30–59 20 1–29  0 0 |
|  | Nicotine  exposure | Self-reported smoking  status | Points: Smoking status:  100 Never smoker  75 Former smoker, quit ≥5 y 50 Former smoker, 1–< 5 y 25 Former smoker, quit <1 y, or currently using inhaled NDS 0 Current smoker |
|  |  |  | Subtract 20 points (unless score is 0) for living with active indoor smoker in home |
|  | Sleep health | Self-reported average  hours of sleep per night | Points: Level (hours) 100 7–<9  90 9–<10  70 6–<7  40 5–<6 or ≥10  20 4–<5  0 <4 |
| Health factors | BMI | Body weight (kilograms)  divided by height squared  (meters squared), and then compare the BMI with the specific values of the same age and sex in the reference table [1]. | Points: BMI (kg/m^2^)  100 <25  70 25.0–29.9  30 30.0–34.9  15 35.0–39.9  0 ≥40.0 |
|  | Blood lipids | Plasma total and HDL  cholesterol with  calculation of non–HDL  cholesterol | Points: Non–HDL cholesterol (mg/dL) 100 <130  60 130–159  40 160–189  20 190–219  0 ≥220 |
|  |  |  | If drug-treated level, subtract 20 points (unless score is 0) |
|  | Blood glucose | Measured fasting blood glucose, HbA1c, and asked participants about their history of diabetes. | Points: Level:  100 No history of T2D and HbA1c <5.7  60 No T2D and HbA1c 5.7–6.4  40 T2D with HbA1c <7.0  30 T2D with HbA1c 7.0–7.9  20 T2D with HbA1c 8.0–8.9  10 T2D with Hb A1c 9.0–9.9  0 T2D with HbA1c ≥10.0 |
|  | Blood pressure | Appropriately measured  systolic and diastolic BPs | Points: Systolic and diastolic BPs (mmHg)  100 <120/<80  75 120–129/<80  50 130–139 or 80–89  25 140–159 or 90–99  0 ≥160 or ≥100 |
|  |  |  | If drug-treated level, subtract 20 points (unless score is 0) |

^[[1]](#footnote-0)^

**References**

1. Centers for Disease Control and Prevention. National Center for Health Statistics, Growth Charts,CDC Growth Charts,Extended BMI-for-Age Charts. Available online: <https://www.cdc.gov/growthcharts/extended-bmi-data-files.htm> (accessed on 8 February 2023).

1. CVH, cardiovascular disease health; BMI, body mass index; T2D, type 2 diabetes. [↑](#footnote-ref-0)
