# Supplementary material for: Investigating how blood cadmium levels influence cardiovascular health scores across sexes and dose responses
Source: Front Public Health. 2024 Aug 21;12:1427905. doi: 10.3389/fpubh.2024.1427905 (PMC11371710; doi:10.3389/fpubh.2024.1427905)
Supplement: Supplementary file 4 [file Table_2.DOCX]

**Table S2** Healthy Eating Index-2015 scoring rules

| Component | Maximum points | Standard for maximum score | Standard for minimum score of zero |
| --- | --- | --- | --- |
| Adequacy |  |  |  |
| Total Fruits | 5 | ≥0.8 cup equivalents/1,000 kcal | No fruits |
| Whole Fruits | 5 | ≥0.4 cup equivalents/1,000 kcal | No whole fruits |
| Total Vegetables | 5 | ≥1.1 cup equivalents/1,000 kcal | No vegetables |
| Greens and Beans | 5 | ≥0.2 cup equivalents/1,000 kcal | No dark green vegetables or Legumes |
| Whole Grains | 10 | ≥1.5 oz equivalents/1,000 kcal | No whole grains |
| Dairy | 10 | ≥1.3 cup equivalents/1,000 kcal | No dairy |
| Total Protein Foods | 5 | ≥2.5 oz equivalents/1,000 kcal | No protein foods |
| Seafood and Plant Proteins | 5 | ≥0.8 cup equivalents/1,000 kcal | No seafood or plant proteins |
| Fatty Acids | 10 | (PUFAs^a^+MUFAs^b^)/SFAs^c^ ≥2.5 | (PUFAs+MUFAs)/SFAs ≤1.2 |
| Moderation |  |  |  |
| Refined Grains | 10 | ≤1.8 oz equivalents/1,000 kcal | ≥4.3 oz equivalents/1,000 kcal |
| Sodium | 10 | ≤1.1 g/1,000 kcal | ≥2.0 g/1,000 kcal |
| Added Sugars | 10 | ≤6.5% of energy | ≥26% of energy |
| Saturated Fats | 10 | ≤8% of energy | ≥16% of energy |

^[[1]](#footnote-0)^

**References**

1. Krebs-Smith SM, Pannucci TE, Subar AF, Kirkpatrick SI, Lerman JL, Tooze JA, Wilson MM, Reedy J (2018) Update of the Healthy Eating Index: HEI-2015. Journal of the Academy of Nutrition and Dietetics 118 (9):1591-1602. doi:10.1016/j.jand.2018.05.021

1. ^a^PUFAs=polyunsaturated fatty acids.

   ^b^MUFAs=monounsaturated fatty acids.

   ^c^SFAs=saturated fatty acids.

   The Healthy Eating Index-2015 (HEI-2015) measures diet quality based on the 2015–2020 Dietary Guidelines for Americans [1]. The HEI-2015 includes 13 components, nine focusing on nutrient adequacy (e.g., total fruit, whole grains, and dairy) and four focusing on moderation (e.g., sodium and saturated fats). Each component has a designated maximum score of 5 or 10 points, which are summed for a maximum score of 100 points, indicating perfect adherence to the Dietary Guidelines for Americans 2015–2020. [↑](#footnote-ref-0)
