# Supplementary material for: Investigating how blood cadmium levels influence cardiovascular health scores across sexes and dose responses
Source: Front Public Health. 2024 Aug 21;12:1427905. doi: 10.3389/fpubh.2024.1427905 (PMC11371710; doi:10.3389/fpubh.2024.1427905)
Supplement: Supplementary file 5 [file Table_3.DOCX]

**Table S3** Baseline characteristics of participants classified by gender

| Characteristic | Total (n = 1658) | Male (n = 1026) | Female (n = 632) | *P* value |
| --- | --- | --- | --- | --- |
| Age^a^ ,years | 49.5 ± 17.4 | 50.4 ± 17.8 | 48.0 ± 16.6 | 0.006 |
| Race^a^ |  |  |  | < 0.001 |
| Mexican American | 160 ( 9.7) | 118 (11.5) | 42 (6.6) |  |
| Non-Hispanic White | 764 (46.1) | 426 (41.5) | 338 (53.5) |  |
| Non-Hispanic Black | 351 (21.2) | 230 (22.4) | 121 (19.1) |  |
| Other race | 383 (23.1) | 252 (24.6) | 131 (20.7) |  |
| Family poverty-income ratio^a^ |  |  |  | 0.156 |
| <1.3 | 432 (26.1) | 255 (24.9) | 177 (28) |  |
| ≥1.3 | 1226 (73.9) | 771 (75.1) | 455 (72) |  |
| Educational level^a^ |  |  |  | 0.005 |
| Below high school | 265 (16.0) | 187 (18.2) | 78 (12.3) |  |
| High-school graduate | 392 (23.6) | 243 (23.7) | 149 (23.6) |  |
| College or above | 1001 (60.4) | 596 (58.1) | 405 (64.1) |  |
| MS, (%)^a^ |  |  |  | < 0.001 |
| Married/living with partner | 967 (58.3) | 632 (61.6) | 335 (53) |  |
| Widowed/divorced/separated | 378 (22.8) | 201 (19.6) | 177 (28) |  |
| Never married | 313 (18.9) | 193 (18.8) | 120 (19) |  |
| Drinking status, (%)^a^ |  |  |  | 0.188 |
| No | 978 (59.0) | 618 (60.2) | 360 (57) |  |
| Yes | 680 (41.0) | 408 (39.8) | 272 (43) |  |
| Waist Circumference, cm^a^ | 100.0 ± 16.1 | 100.6 ± 15.5 | 99.1 ± 17.0 | 0.074 |
| eGFR, mL/min/1.73m^2 a^ | 96.7 ± 22.9 | 89.6 ± 21.5 | 108.3 ± 20.2 | < 0.001 |
| log Cd, log μg/dL^a,b^ | -0.837 ± 0.864 | -0.938 ± 0.864 | -0.673 ± 0.838 | < 0.001 |
| Body mass index scores^a^ | 59.7 ± 33.2 | 62.4 ± 31.0 | 55.3 ± 36.1 | < 0.001 |
| Blood pressure scores^a^ | 64.3 ± 34.3 | 61.1 ± 33.8 | 69.5 ± 34.5 | < 0.001 |
| Blood lipids scores^a^ | 66.2 ± 30.5 | 65.7 ± 30.2 | 67.1 ± 31.0 | 0.349 |
| Blood glucose scores^a^ | 80.6 ± 26.7 | 78.6 ± 27.6 | 84.0 ± 24.8 | < 0.001 |
| Physical activity scores^a^ | 90.8 ± 18.2 | 92.0 ± 17.3 | 88.9 ± 19.4 | < 0.001 |
| Nicotine exposure scores^a^ | 38.7 ± 34.9 | 39.0 ± 35.0 | 38.1 ± 34.7 | 0.62 |
| Sleep health scores^a^ | 82.5 ± 24.6 | 81.6 ± 25.2 | 83.9 ± 23.6 | 0.066 |
| Diet scores^a^ | 49.9 ± 41.0 | 48.2 ± 41.1 | 52.6 ± 40.9 | 0.036 |
| CVH scores^a^ | 66.6 ± 12.9 | 66.1 ± 12.2 | 67.4 ± 13.9 | 0.038 |

^[[1]](#footnote-0)^

1. CVH cardiovascular health.

   ^a^ Continuous variables are presented as mean ± SD; categorical variables are presented as N(%).

   ^b^ Those values retains three decimal places. [↑](#footnote-ref-0)
