# Supplementary material for: Investigating how blood cadmium levels influence cardiovascular health scores across sexes and dose responses
Source: Front Public Health. 2024 Aug 21;12:1427905. doi: 10.3389/fpubh.2024.1427905 (PMC11371710; doi:10.3389/fpubh.2024.1427905)
Supplement: Supplementary file 6 [file Table_4.DOCX]

**Table S4** Threshold effect analysis of the relationship between the blood Cd concentrations and CVH scores

| log Cd (log μg/dL) | Adjusted Model | |
| --- | --- | --- |
|  | β (95% CI) | *P* value |
| <-1.447  ≥-1.447 | -0.648 (-2.811-1.516)  -4.866(-5.938--3.794) | 0.557  <0.001 |
| Logarithmic likelihood ratio test 𝑃 value |  | <0.001 |

^[[1]](#footnote-0)^

1. CVH cardiovascular health ; Adjusted Model was adjusted for age, sex, race, family PIR, educational level, marital status, drinking status, waist circumference, and eGFR. Only 95% of the data is displayed. [↑](#footnote-ref-0)
