# Supplementary material for: Investigating how blood cadmium levels influence cardiovascular health scores across sexes and dose responses
Source: Front Public Health. 2024 Aug 21;12:1427905. doi: 10.3389/fpubh.2024.1427905 (PMC11371710; doi:10.3389/fpubh.2024.1427905)
Supplement: Supplementary file 8 [file Table_6.DOCX]

**Table S6** Association between the overall and component CVH scores and blood Cd levels after excluded subjects with baseline cardiovascular disease

|  |  | CVH | | Body mass index | | Blood pressure | | Blood lipids | | Blood glucose | | Physical activity | | Nicotine exposure | | Sleep health | | Diet | |
| --- | --- | --- | --- | --- | --- | --- | --- | --- | --- | --- | --- | --- | --- | --- | --- | --- | --- | --- | --- |
|  |  | Model 1 | Model 2 | Model 1 | Model 2 | Model 1 | Model 2 | Model 1 | Model 2 | Model 1 | Model 2 | Model 1 | Model 2 | Model 1 | Model 2 | Model 1 | Model 2 | Model 1 | Model 2 |
| Cd |  | | | | | | | | | | | | | | | | | | |
| Continuous | 1475 | -2.81 (-3.56~-2.07)* | -3.27 (-3.92~-2.61)* | 3.18 (1.23~5.12)* | 0.33 (-0.76~1.43) | 0.41 (-1.59~2.41) | 0.43 (-1.42~2.29) | 0.5 (-1.32~2.32) | -1.26 (-3.11~0.58) | 1.01 (-0.51~2.53) | 0.39 (-1.04~1.82) | -0.01 (-1.05~1.04) | 0.34 (-0.75~1.44) | -19.67 (-21.43~-17.92)* | -19.09 (-20.61~-17.57)* | -2.53 (-3.94~-1.12)* | -2.09 (-3.56~-0.63)* | -5.4 (-7.79~-3.01)* | -5.18 (-7.56~-2.81)* |
| Q1 | 367 | Ref | Ref | Ref | Ref | Ref | Ref | Ref | Ref | Ref | Ref | Ref | Ref | Ref | Ref | Ref | Ref | Ref | Ref |
| Q2 | 358 | 0.67 (-1.17~2.5) | -0.37 (-1.93~1.18) | 3.36 (-1.47~8.2) | 0.3 (-2.3~2.91) | 1.33 (-3.63~6.29) | 4.71 (0.3~9.11)* | -0.7 (-5.21~3.82) | -0.48 (-4.87~3.91) | 0.37 (-3.4~4.14) | 2.17 (-1.22~5.57) | -1.17 (-3.77~1.44) | -0.54 (-3.14~2.07) | -7.55 (-11.83~-3.28)* | -13.29 (-16.91~-9.68)* | 2.39 (-1.1~5.89) | 1.19 (-2.3~4.67) | 7.29 (1.39~13.18)* | 2.94 (-2.69~8.57) |
| Q3 | 385 | -2.27 (-4.08~-0.47)* | -3.96 (-5.52~-2.4)* | 7.5 (2.75~12.25)* | -0.25 (-2.88~2.38) | -2.37 (-7.24~2.5) | 0.89 (-3.55~5.33) | 1.61 (-2.82~6.04) | -0.56 (-4.99~3.87) | 1.03 (-2.67~4.73) | 2.13 (-1.29~5.55) | -0.14 (-2.7~2.42) | 0.61 (-2.02~3.24) | -21.37 (-25.57~-17.17)* | -25.34 (-28.99~-21.69)* | -1.36 (-4.79~2.08) | -1.67 (-5.19~1.84) | -3.09 (-8.88~2.7) | -7.49 (-13.17~-1.81)* |
| Q4 | 365 | -6.37 (-8.19~-4.54)* | -7.38 (-8.98~-5.78)* | 7.53 (2.71~12.34)* | 0.93 (-1.76~3.62) | 2.12 (-2.81~7.06) | 1.04 (-3.5~5.59) | 1.4 (-3.09~5.89) | -2.61 (-7.15~1.93) | 3.02 (-0.73~6.77) | 1.04 (-2.46~4.55) | -0.27 (-2.86~2.33) | 0.55 (-2.14~3.24) | -47.89 (-52.15~-43.64)* | -45.63 (-49.36~-41.89)* | -4.96 (-8.44~-1.49)* | -3.7 (-7.3~-0.11)* | -11.87 (-17.73~-6)* | -10.69 (-16.5~-4.87)* |

^[[1]](#footnote-0)^

1. CVH: cardiovascular health; Model 1 was crude model; Model 2 was adjusted for age, sex, race, family PIR, educational level, marital status, drinking status, waist circumference, and eGFR.

   ^*^: *P* < 0.05. [↑](#footnote-ref-0)
