# Supplementary material for: Investigating how blood cadmium levels influence cardiovascular health scores across sexes and dose responses
Source: Front Public Health. 2024 Aug 21;12:1427905. doi: 10.3389/fpubh.2024.1427905 (PMC11371710; doi:10.3389/fpubh.2024.1427905)
Supplement: Supplementary file 9 [file Table_7.DOCX]

**Table S7** Baseline characteristics stratified by blood Cd quartiles

| Variables | Total (n = 1658) | Q1 (n = 398) | Q2 (n = 413) | Q3 (n = 430) | Q4 (n = 417) | *P* value |
| --- | --- | --- | --- | --- | --- | --- |
| Total fruit^a^ | 0.4 ± 0.5 | 0.4 ± 0.5 | 0.4 ± 0.6 | 0.4 ± 0.6 | 0.2 ± 0.4 | < 0.001 |
| Whole fruit^a^ | 0.5 ± 0.6 | 0.5 ± 0.6 | 0.6 ± 0.6 | 0.5 ± 0.6 | 0.4 ± 0.5 | < 0.001 |
| Total vegetables^a^ | 0.8 ± 0.6 | 0.8 ± 0.6 | 0.8 ± 0.6 | 0.8 ± 0.6 | 0.7 ± 0.6 | 0.041 |
| Greens and Beans^a^ | 0.1 ± 0.2 | 0.1 ± 0.2 | 0.2 ± 0.3 | 0.1 ± 0.3 | 0.1 ± 0.2 | 0.014 |
| Whole grains^a^ | 0.5 ± 0.6 | 0.4 ± 0.6 | 0.6 ± 0.7 | 0.5 ± 0.6 | 0.4 ± 0.6 | < 0.001 |
| Dairy^a^ | 0.7 ± 0.5 | 0.7 ± 0.5 | 0.7 ± 0.5 | 0.6 ± 0.5 | 0.7 ± 0.5 | 0.32 |
| Meat and beans^a^ | 3.3 ± 1.7 | 3.4 ± 1.7 | 3.4 ± 1.7 | 3.2 ± 1.5 | 3.0 ± 1.7 | 0.004 |
| Seafood and Plant Proteins^a^ | 0.8 ± 1.2 | 0.8 ± 1.1 | 1.0 ± 1.3 | 0.8 ± 1.1 | 0.7 ± 1.3 | 0.053 |
| Fatty Acids^a^ | 1.9 ± 0.6 | 1.9 ± 0.6 | 2.0 ± 0.6 | 1.9 ± 0.7 | 1.8 ± 0.6 | 0.027 |
| Refined Grains^a^ | 2.6 ± 1.2 | 2.7 ± 1.2 | 2.5 ± 1.3 | 2.5 ± 1.3 | 2.5 ± 1.1 | 0.048 |
| Sodium^a^ | 1.7 ± 0.5 | 1.7 ± 0.4 | 1.7 ± 0.4 | 1.7 ± 0.5 | 1.7 ± 0.5 | 0.363 |
| Added Sugars^a^ | 11.7 ± 8.0 | 10.2 ± 6.5 | 10.0 ± 6.3 | 11.8 ± 7.4 | 14.6 ± 10.1 | < 0.001 |
| Saturated fats^a^ | 11.4 ± 3.4 | 11.4 ± 3.2 | 11.4 ± 3.5 | 11.4 ± 3.4 | 11.3 ± 3.4 | 0.961 |

^[[1]](#footnote-0)^

1. ^a^ Continuous variables are presented as mean ± SD; Q1: Cd < -1.4 log μg/dL; Q2: -1.4–-0.8 log μg/dL; Q3: -0.8–-0.2 log μg/dL; Q4: Cd ≥ -0.2 log μg/dL. [↑](#footnote-ref-0)
